# 668. Single-stage Full-thickness Grafting with a Novel Method for Skin Graft Fixation for Post-burn Hand Contractures

**DOI:** 10.1093/jbcr/irag033.534

**Published:** 2026-04-06

**Authors:** Tz-Wei Chiou, Cheng-I Yen, Shiow-Shuh Chuang

**Affiliations:** Chang Gung Memorial Hospital, New Taipei City, New Taipei; Chang Gung Memorial Hospital, New Taipei City, New Taipei; Chang Gung Memorial Hospital, Taoyuan City, Taoyuan

## Abstract

**Introduction:**

Post-burn hand contractures frequently result in functional impairment and aesthetic deformity. Traditional staged surgical approaches often require multiple procedures and carry a high risk of recurrent contractures. Full-thickness skin grafting (FTSG) is a recognized method for reconstruction, but securing grafts over complex hand anatomy with tie-over bolster dressings can be time-consuming and technically challenging, especially when multiple fingers are involved. This study evaluated the outcomes of one-stage scar release with FTSG in combination with a novel graft fixation method using self-adherent elastic wrap for complex post-burn hand contractures.

**Methods:**

We retrospectively reviewed patients between 2016 and 2023 who had deep second- or third-degree hand burns (>20% TBSA), were aged ≥18 years, and had at least 6 months of follow-up. All patients underwent one-stage scar excision and FTSG for post-burn hand contractures, including claw hand, syndactyly, and webspace contractures. FTSG over the fingers was secured using self-adherent elastic wrap instead of traditional tie-over dressing. Outcomes assessed included range of motion (ROM) of the wrist and digits, graft take, postoperative complications, donor-site morbidity, and follow-up duration.

**Results:**

A total of 57 patients (85 hands; mean age 23 years, mean TBSA 59.5%) were included. Contractures involved the dorsum (n = 79) and flexion deformities (n = 6). Postoperatively, mean ROM improved from 56.1° to 123.9°, and functional wrist ROM increased from 42.5% to 93.2% (*p*<.01). Graft take exceeded 95% in all but one case. No significant hematoma, necrosis, or infection was observed with the self-adherent wrap method, and donor-site morbidity was minimal. No patient required secondary arthrolysis during a mean follow-up of 24 months (range 7–78 months). The use of self-adherent wrap simplified graft immobilization, reduced operative time, and provided reliable outcomes for complex hand reconstruction.

**Conclusions:**

One-stage scar release with FTSG, combined with self-adherent elastic wrap fixation, is a safe, efficient, and effective approach for complex post-burn hand contractures. This method achieves durable functional restoration, minimizes recurrence, and overcomes technical challenges associated with conventional tie-over dressings, offering both functional and aesthetic benefits while reducing operative time.

**Applicability of Research to Practice:**

This study demonstrates that one-stage scar release with full-thickness skin grafting (FTSG) effectively restores function and aesthetics in post-burn hand contractures. Using a self-adherent elastic wrap simplifies graft fixation, shortens operative time, enables early rehabilitation, and reduces the need for multiple surgeries.

**Funding for the study:**

N/A.

